# Cold-Start Problems in Data-Driven Prediction of Drug–Drug Interaction Effects

**DOI:** 10.3390/ph14050429

**Published:** 2021-05-02

**Authors:** Pieter Dewulf, Michiel Stock, Bernard De Baets

**Affiliations:** KERMIT, Department of Data Analysis and Mathematical Modelling, Ghent University, Coupure Links 653, 9000 Gent, Belgium; michiel.stock@ugent.be (M.S.); bernard.debaets@ugent.be (B.D.B.)

**Keywords:** polypharmacy, drug–drug interaction, prediction, cross-validation, machine learning, cold-start problems

## Abstract

Combining drugs, a phenomenon often referred to as polypharmacy, can induce additional adverse effects. The identification of adverse combinations is a key task in pharmacovigilance. In this context, in silico approaches based on machine learning are promising as they can learn from a limited number of combinations to predict for all. In this work, we identify various subtasks in predicting effects caused by drug–drug interaction. Predicting drug–drug interaction effects for drugs that already exist is very different from predicting outcomes for newly developed drugs, commonly called a cold-start problem. We propose suitable validation schemes for the different subtasks that emerge. These validation schemes are critical to correctly assess the performance. We develop a new model that obtains AUC-ROC =0.843 for the hardest cold-start task up to AUC-ROC =0.957 for the easiest one on the benchmark dataset of Zitnik et al. Finally, we illustrate how our predictions can be used to improve post-market surveillance systems or detect drug–drug interaction effects earlier during drug development.

## 1. Introduction

Interaction with targets such as proteins, DNA, etc. enables a drug to combat diseases. Interaction with off-targets, i.e., interaction that is initially not intended, can lead to additional effects. These effects can either be positive or adverse, but are considered as side effects when judged secondary to the main therapeutic effect. Accurate and efficient predictions for the interactions can accelerate the drug development process and help to obtain lower attrition rates [1]. Safety issues can pop up during the drug development process up until the clinical trials, after which a drug can enter the market [2,3]. At this stage, harmful adverse effects are quite rare, however, they still are an important cause of morbidity and mortality [4,5]. Estimates range from 100,000 to almost 200,000 fatalities in the US and Europe, respectively, making them the fourth cause of death before pulmonary diseases and diabetes [3,6].

Combining drugs can be a useful, effective and sometimes indispensable strategy to combat certain diseases [7,8,9,10]. It is suited to interact with multiple targets at the same time and may improve the efficacy of a therapy. However, interaction between drugs increases the risk of additional adverse effects [9,11,12]. Therefore, polypharmacy, often defined as the administration of five or more medications daily, is typically regarded as undesirable. Around 2010–2015, polypharmacy was recognized as a highly increasing phenomenon [13], affecting about 15% of the US population [12]. Mainly older adults frequently have several chronic health conditions requiring multiple medications; 66.8% of 65 years and older US citizens take three or more prescription medications [14,15].

Pharmacovigilance systems have been created for post-market surveillance to facilitate monitoring and support of regulatory action against harmful adverse effects [16]. A prominent example is the FDA Adverse Event Reporting System (FAERS), collecting adverse event reports that were submitted to the FDA by healthcare professionals, consumers, and manufacturers. Various online tools, e.g., https://www.drugs.com/drug_interactions.html (accessed on 1 May 2021), https://go.drugbank.com/interax/multi_search (accessed on 1 May 2021), based on such systems enable users to search for interacting combinations themselves. These systems, however, are unlikely to contain every possible effect for every possible combination of drugs.

The trend of combining drugs has led to a growing research interest in drug–drug interaction, both for the search for effective combinations as well as for adverse effects. However, due to high number of combinations, systematic screening in in vitro experiments and clinical trials is challenging [9,11,17,18,19,20,21]. Alternatively, machine learning approaches have been proposed that can learn from a limited number of drug–drug interactions to predict the effects for all drug–drug combinations. For example, Zitnik et al. [22] were the first to use data extracted from adverse event reporting systems to predict the specific adverse effects associated with drug–drug combinations. As Zitnik et al. published the data used, other researchers have continued tackling the same problem, increasing the predictive accuracy [23,24,25,26]. In addition, various machine learning approaches were recently presented to identify positive effects of combining existing drugs. This includes, for example, predicting synergies between anticancer drugs in particular cell lines, or directly predicting for which disease a drug–drug combination is effective [27,28,29].

In this work, we first formulate the drug–drug interaction effect prediction problem in a general way (Section 2.1). We will focus on adverse effects, also called adverse drug reactions (ADR), in the context of pharmacovigilance; however, the formulation is valid for modeling any type of effect, whether positive or adverse. Crucial in our formulation is distinguishing between various prediction tasks. The different tasks have a different level of “cold start”. In a cold-start prediction task in general, less information is available for prediction due to the introduction of a new entity. A typical example is when a new user creates a Netflix account and one wants to recommend new movies without knowing which other movies are liked by the user. Cold-start tasks are often more difficult. A similar reasoning holds when one wants to predict whether a certain effect is linked to combining two drugs: the task differs on whether other effects caused by this pair are known or not, or whether effects of one of the two drugs in combination with other drugs are known or not, etc. Based on such reasoning, we distinguish between four prediction tasks. Two of these tasks may serve to improve pharmacovigilance systems by detecting drug–drug interaction effects in combinations with existing drugs. Two other tasks rather take into account a new drug for which no interaction is a priori known: this could be useful to foresee drug–drug interaction effects even already during development.

In Section 2.2, we discuss how one can validate models for the different tasks. An important aspect of our work is using the proper cross-validation scheme that reflects the desired task. For instance, given the available data, if we want to validate the model on the task of predicting for a “new drug”, we must make sure that any interaction data we have on this drug is used for model validation, and never for training the model. This corresponds to simulating and validating predictions for a new drug without actually needing clinical tests with new drugs. Additionally, we discuss two ways to evaluate a performance metric, one that focuses on predicting the right interacting drug–drug pairs and another one that focuses on predicting the right effects.

In Section 2.3, we introduce the data set on pharmacovigilance presented by Zitnik et al. [22], originating from an adverse event reporting system. It contains molecules used in medications and various adverse effects caused by combining two compounds in a treatment, ranging from nausea, headache, or nightmare to acute kidney failure, lung fibrosis, heart attack, or still birth. We propose a new model called three-step kernel ridge regression, an extension of two-step kernel ridge regression [30], that can tackle the different tasks. We train and validate the model on the data, according to the results presented in Section 2.2. Further, we show how these results and tasks are related to the work of Zitnik et al. [22].

We stress that the model predictions for adverse effects due to drug–drug interactions are not the ground truth. The more accurate the model, the lower the risk of making a wrong prediction, however, there is no guarantee that the model would not over-detect adverse reactions. The model can be seen as a supporting tool, and, for example, could be used to detect interesting combinations that could be further investigated by experts or in clinical tests. In Section 2.4, we provide an illustration on how predictions could be used regarding the different tasks, by selecting the strongest model predictions and visualizing them.

## 2. Results

### 2.1. Formulation of the Prediction Subtasks

Consider a set of drugs $D=\{d_{i}|i=1,\ldots ,n_{d}\}$ that are on the market and a set of possible effects $E=\{e_{i}|i=1,\ldots ,n_{e}\}$. We assign a label $Y_{ijk}=1$ if the interaction between drug $d_{i}$ and drug $d_{j}$ can cause effect $e_{k}$ and a label $Y_{ijk}=0$ if not. Note that we only consider effects due to interaction and do not take into account effects caused by one of the drugs individually. We consider the general problem of predicting the label for any combination of two drugs and for any effect by modeling a prediction function:

$$f:(d,d^{\prime },e)\mapsto r\;.$$

The larger the predicted value r∈R, the more likely the effect is linked to the interaction between the two drugs. These values can be used to rank the effects, and, if necessary, be mapped to probability scores in the unit interval [0,1] [31]. The function is learned on the basis of labeled data. We subdivide the problem into different prediction tasks. The tasks defined next differ both in practical applicability and difficulty:

1. $\hat{dde}$: unknown drug–drug-effect. Predict the occurrence of an effect for a drug–drug pair for which other effects are already known. This problem corresponds to regular tensor completion problems in machine learning.
2. $\hat{dd}e$: unknown drug–drug pair. Predict for a drug–drug pair for which no interaction effect is known. This is the first cold-start task.
3. $\hat{d}de$: unknown drug. Predict for a new drug for which no effect is known in any combination with another drug. This is the second cold-start task.
4. $\hat{d}\hat{d}e$: two unknown drugs. Predict for two new drugs for which no effect is known in any combination with another drug. This is the third cold-start task.

**Figure 1:**
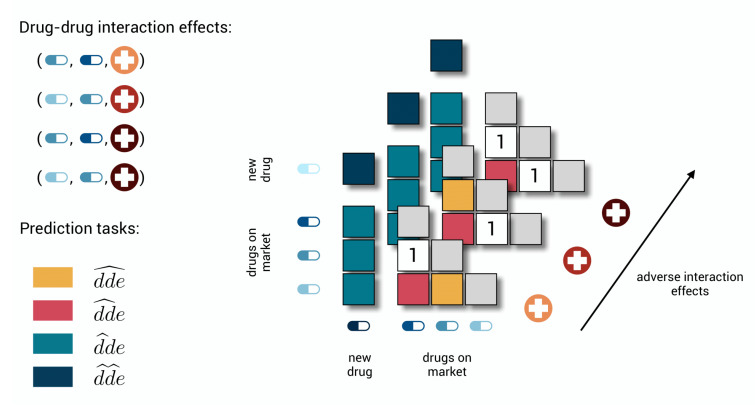
The different tasks in a toy polypharmacy prediction problem. There are three possible adverse effects and three drugs on the market for which two different pairwise combinations are labeled to cause two specific adverse effects. Because of symmetry, it is sufficient to only store values on one side of the diagonal, as the other side is only representing the same combinations with the drugs interchanged. In the first task, the goal is to predict whether also the third specific adverse effect would occur for the two combinations that already have some known effects. In the second, the goal is to predict whether and which effects would occur for the third combination of on market drugs for which no effect is known. In task three, a new drug is added for which no label can yet be found, and one wants to predict whether drug–drug interaction effects would occur in combination with the drugs that are already on the market. In the last task, the goal is to predict whether and which effects would occur when two new drugs are combined.

The subtasks are illustrated in Figure 1, focusing on adverse effects in the context of pharmacovigilance. The drug–drug-effect triplets represent drug–drug interaction effects extracted from post-market surveillance systems. Note that we are not assigning a specific role to the order of the drugs, i.e., $Y_{ijk}=Y_{jik}$, while a drug obviously cannot interact with itself, i.e., $Y_{iik}=0$.

The first two tasks can serve to improve the post-market surveillance system. It is unlikely that every possible effect for every possible combination is significantly represented by reports in the system. If the model predicts a high value for a drug–drug-effect triplet, while that triplet could not be extracted from the system and a zero-label was initially assigned, then it is likely that a new drug–drug interaction effect has been detected in silico. Whereas a task-one model focuses on detecting additional adverse effects for a drug–drug pair if some other adverse effects are already known, a task-two model needs to predict the occurrence of all adverse effects for that pair from scratch.

The third task is relevant for both the drug development process and the post-market surveillance systems. It reflects the situation where a new drug enters the market or a drug is under development, and one wants to foresee the possible adverse effects in combination with drugs that are already on the market. Again, combinations with the highest predictions are most likely to occur and could be further investigated by experts or tested in clinical trials. The fourth task is added for the sake of completeness but may be relevant to drug development as well. For example, for a complex disease where one needs to act on multiple targets, it may be easier to develop a treatment with two new drugs rather than searching for one that acts on all targets.

The tasks are of increasing difficulty. Each time when the model needs to predict for a drug–drug-effect triplet, less information is available for the model to learn the interaction. From the first to the second task, information on other effects for the drug–drug pair disappears, making it the first cold-start problem. The third and fourth tasks are more difficult cold-start problems because any information on one of the drugs or both drugs disappears, respectively (cf. zero-shot problems [32]). The notation for the respective prediction tasks suggests that even more prediction tasks could be distinguished, such as $dd\hat{e}$, corresponding to the introduction of a new effect. However, at this moment, we believe that the four ones discussed constitute more relevant real-life problem settings.

### 2.2. Validation Procedures for the Prediction Subtasks

**Figure 2:**
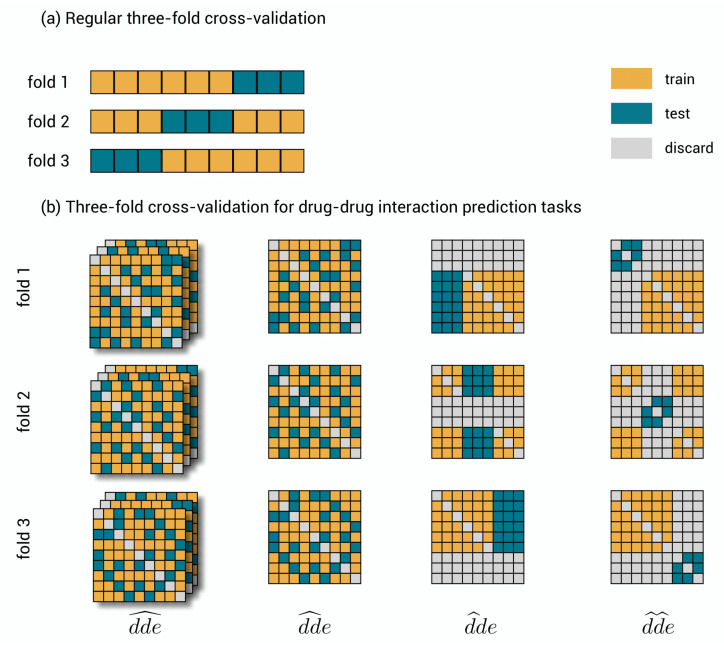
Cross-validation. (**a**) Three-fold cross-validation for a traditional data set. The data set is shown three times, where each time another chunk is used for evaluating the model and the remaining part for training. (**b**) Three-fold cross-validation for the polypharmacy prediction tasks. A symmetric toy data set of nine by nine drug–drug-pairs and three distinct effects. In $\hat{dde}$, triplets are randomly, but symmetrically assigned to one of the folds. In $\hat{dd}e$, drug–drug pairs are randomly but symmetrically assigned to one of the folds. As this assignment is the same for each effect, only one slice is shown. In $\hat{d}de$, drugs are as a whole assigned to one of the folds. Note that the symmetric counterpart is to be discarded. This assignment is again the same for each effect slice. In $\hat{d}\hat{d}e$, drug–drug-pairs are as a whole assigned to one of the folds, but now both drugs need to be test drugs and any other interaction is to be discarded.

Cross-validation is the standard approach for the validation of machine learning models [33]. A usual data set $\left\{\left(x_{i},y_{i}\right)|i=1,2,\ldots ,n\right\}$ consists of objects $x_{i}\in X$ and corresponding labels $y_{i}\in Y$. This data set is split into a training set and a test set. The training set can be used to learn the model, while the test set must remain unused during learning and serves to evaluate the predictions of the model using a performance metric. To account for statistical fluctuations related to the specific realization of the split, this procedure is repeated *k* times such that each object and its label are exactly once part of the test set. This approach is called *k*-fold cross-validation; the *k* folds are easily obtained by dividing the data set into *k* equal chunks of size n/k, each time using one chunk as test set. Subsequently, one can pool the test predictions of the different test sets for evaluation or evaluate separately and average. An example of 3-fold cross-validation is shown in Figure 2a.

The objects in our problem setting are triplets $\left(d_{i},d_{j},e_{k}\right)$ and three indices are needed to denote a specific label $Y_{ijk}$. Mathematically, the triplet labels build up a tensor, a three-dimensional generalization of a matrix. The three-dimensional structure of the tensor makes the process of dividing the data into chunks somewhat more complicated, though interesting. There are several ways to perform cross-validation. We present four ways that reflect the different prediction tasks. The different cross-validation schemes are visualized in Figure 2b on the full symmetric tensor (i.e., $Y_{ijk}$ = $Y_{jik}$), allowing to visualize the consequences of symmetry. They are described as follows:

1. $\hat{dde}$: drug–drug-effect triplets are randomly assigned to the different test sets. Performance for a triplet is thus measured without any restriction on the availability of other triplets in the training data.
2. $\hat{dd}e$: drug–drug pairs are randomly assigned to the different test sets together with all the effects. Performance is thus measured with the restriction that for the drug–drug pair of a test triplet, not a single link with an effect is part of the training data.
3. $\hat{d}de$: the first drugs are randomly assigned to the different test sets, together with all combinations with all other drugs and all effects. Performance is thus measured with the restriction that for the first drug of a test triplet, not a single effect from interaction with any other drug is part of the training data.
4. $\hat{d}\hat{d}e$: drugs are assigned to the different test sets, at the same time for the first and second drug and for all effects. Prediction is thus measured with the restriction that for both drugs of a test triplet, not a single effect from interaction with any other drug is part of the training data.

The performance highly depends on the cross-validation scheme. Therefore, it is important for model validation and tuning to use the proper scheme that reflects the task under consideration.

**Figure 3:**
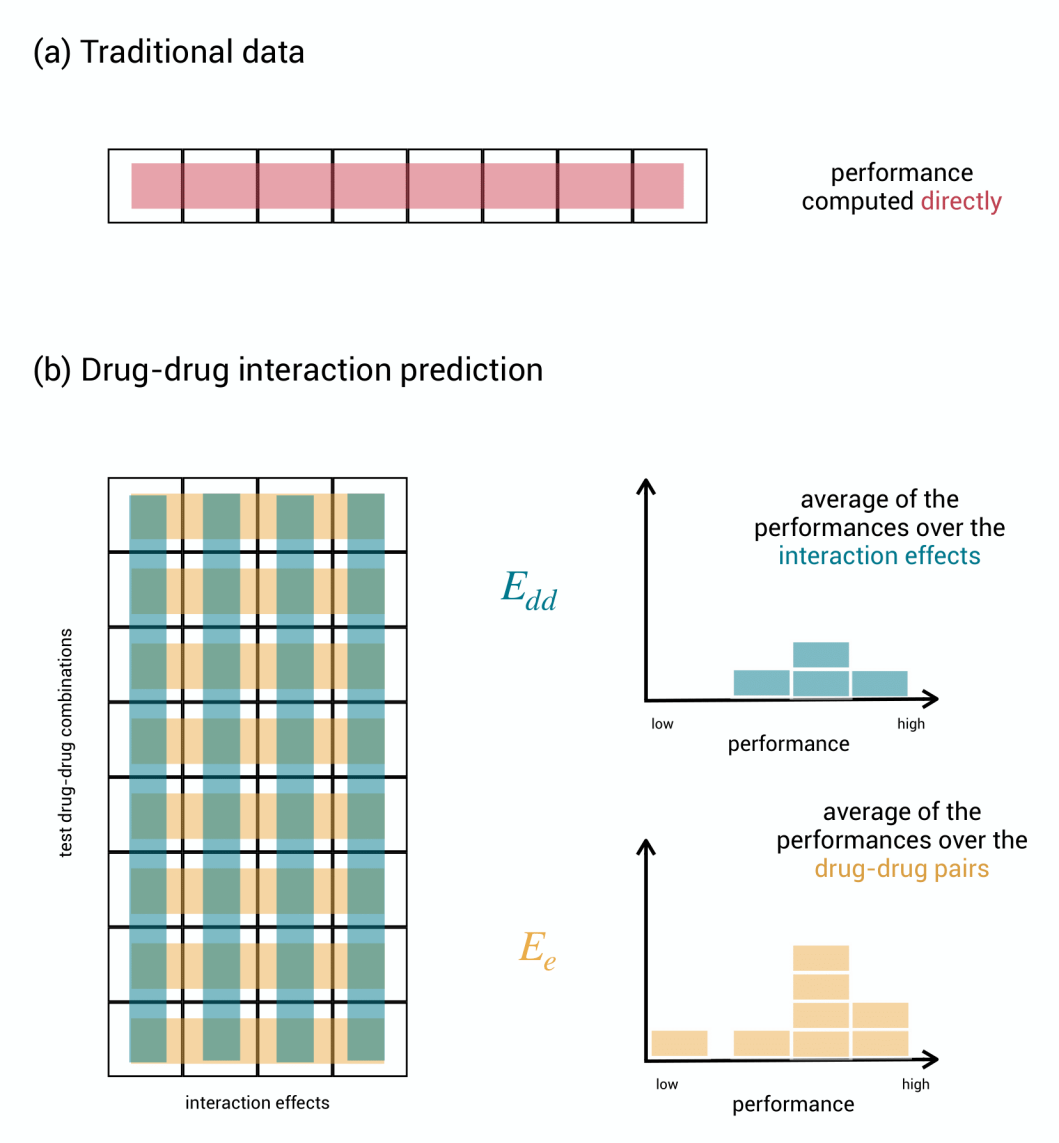
Evaluation schemes. (**a**) Regular, unstructured data where performance is computed directly by comparing the true test labels to the predicted test labels. (**b**) Aggregation of computations of performance in the matrix of test drug–drug pairs versus effects. In evaluation scheme $E_{dd}$, for each effect, the performance measures how well the model can discriminate between drug–drug pairs. The $E_{dd}$ performances for the different effects can be gathered in a histogram and averaged to obtain a single final performance. In evaluation scheme $E_{e}$, for each drug–drug pair, the performance measures how well the model can discriminate between effects. The $E_{e}$ performances for the different drug–drug pairs can be gathered in a histogram and averaged to obtain a single final performance.

As the considered prediction tasks are binary classification tasks, i.e., predicting whether a drug–drug interaction effect occurs (1) or not (0), typical classification performance metrics such as AUC-ROC or AUC-PR can be used for expressing predictive performance. This is done straightforwardly for regular classification problems: one calculates the performance metric on the list of predicted test labels by comparing them to the true test labels, and this immediately yields the final performance result, as shown in Figure 3a.

The three-dimensional nature of our problem, however, opens up various approaches to evaluate performance. Here, we consider two evaluation schemes with a different focus and a corresponding relevant interpretation. Before getting started, we simplify the presentation by collecting the test labels into a matrix with drug–drug pairs as rows and effects as columns and, as shown in Figure 3b.

In the first scheme, $E_{dd}$, the performance metric is calculated for each column separately and then averaged. Each column involves one effect and covers the comparison of the predicted and the true labels on all test drug–drug pairs. A high column performance means that the model can well discriminate between interacting and non-interacting drug–drug pairs for the given effect. In the second scheme, $E_{e}$, the performance metric is calculated for each row separately and then averaged. Each row involves one drug–drug pair and covers the comparison of the predicted and the true labels on the various effects. A high row performance now means that the model is well able to specify which effects are likely to occur given the drug–drug pair. Summarizing, the performance in scheme $E_{dd}$ rather expresses the capacity of the model to discriminate between interacting and non-interacting drug–drug pairs on average, while the performance in scheme $E_{e}$ rather expresses the capacity to predict the right effects on average.

Consider a hypothetical model that predicts all the effects as occurring for each drug–drug pair that has at least one effect in the database. On the data published by Zitnik et al. [22], this model obtains AUC-ROC_*dd*_ =0.85 for identifying interacting drug–drug pairs and a poor AUC-ROC_*e*_ =0.5 for assigning the right adverse effects. This is because when two drugs interact, often a larger set of effects occurs. Predicting that all effects occur does affect $E_{dd}$ performance only a bit, while $E_{e}$ performance indicates the model to be completely useless in predicting the right effects. We propose to take both evaluation schemes into account as predictions that are good in one scheme are not guaranteed to be good in the other scheme.

By averaging the individual performances into a single score, information on the distribution of performance may be lost; some effects may be much harder to predict for than others, and one would not know it. Therefore, we propose not to neglect the distribution of performance when judging a model, as shown in Figure 3b.

### 2.3. Model Training and Validation

We perform our experiments and analysis on the data set published by Zitnik et al. [22], which contains drug–drug interaction effects extracted from adverse event reporting systems. This data set contains 645 drugs and 963 adverse effects, with 2% of the possible triplets being occurring triplets with a one-label caused by 70% of the drug–drug pairs. Any other triplet is assumed to have a zero-label. We also take side information on the drugs and effects into account, which is important for cold-start tasks when no label information is available. For the drugs, we use the single-drug effects published by Zitnik et al. [22]. These indicate on which targets the individual drugs are acting. By combining the target information of both drugs, the model can better learn when an interaction occurs. For the effects, we use training labels in order to construct features. More details can be found in the Materials and Methods section.

We introduce a new model, called three-step kernel ridge regression, that can tackle all prediction tasks in a unified way. This model is a natural generalization of two-step kernel ridge regression [30,34,35] for pairwise interactions. The model can take any side information on the drugs and the effects into account. The difference between the prediction tasks is accounted for by three regularization parameters. These are the hyper-parameters of the model and can be tuned for optimal performance on a specific task. Further, this model is extremely efficient and provides algebraic shortcuts for fast cross-validation and tuning of the regularization parameters. These shortcuts are a generalization of the shortcuts for the two-step model [36]. More details can be found in the Materials and Methods section and in Appendix A.

**Figure 4:**
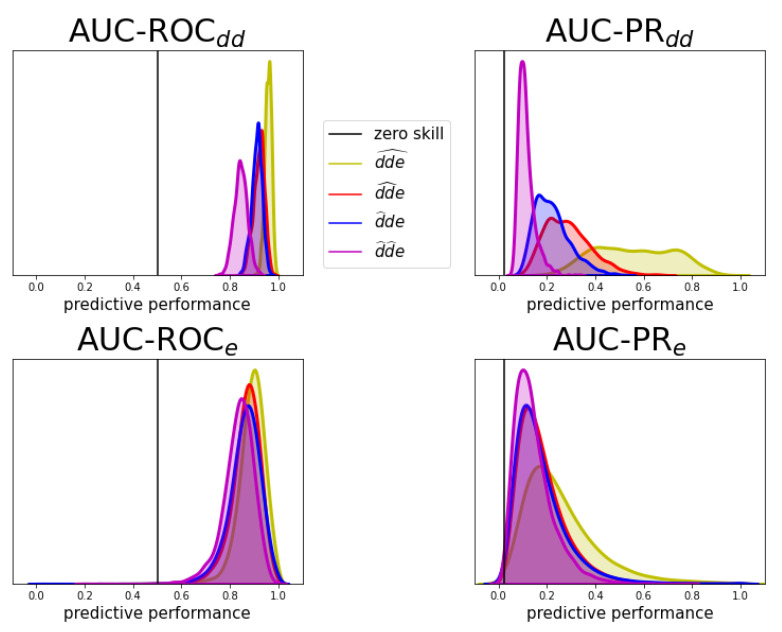
Normalized performance distributions for the different prediction tasks, obtained as illustrated in Figure 3 for both evaluation schemes. The test predictions of the ten folds were pooled together into one final evaluation such that each distribution is unambiguously determined. No-skill performance for AUC-ROC and AUC-PR is 0.5 and 0.02, respectively. Details on the computation and the no-skill value of AUC-PR can be found in Appendix D. Both metrics have perfect-skill performance at 1.

We perform 10-fold cross-validation with the three-step model for each of the prediction tasks for both evaluation schemes and both the AUC-ROC and AUC-PR performance metrics. The predictive performances are shown by distribution in Figure 4 and by average value in Table 1, as was introduced in Figure 3. The AUC scores provide a quick view on how well a model is performing. However, note that a perfect AUC score of 1 can only be obtained with a proper predictive model if also the data is correctly labeled. We expect a certain percentage of false negative labels, i.e., occurring adverse effects due to drug–drug interaction that could not be extracted from the surveillance system. Especially for prediction tasks one and two, we need a suboptimal AUC to be able to detect them.

The expected order in difficulty (i.e., $\hat{dde}$ followed by $\hat{dd}e$, $\hat{d}de$, and $\hat{d}\hat{d}e$) is reflected in a descending performance in both evaluation schemes and for both performance metrics. The drop in performance may seem harder for $E_{dd}$ than for $E_{e}$. This effect can be explained by the fact that as new drugs or new drug combinations enter through the different tasks, the model has more difficulties with distinguishing between the drug pairs rather than between the effects (which remain the same). The performance distribution for identifying interacting drug–drug pairs (evaluation scheme $E_{dd}$) in the upper row of Figure 4 shows that correctly detecting adverse pairs may be easier for one effect than for another. Still, performance is almost always substantially better than a random baseline, even for the most difficult cold-start prediction task $\hat{d}\hat{d}e$. Equally, the performance distribution for assigning the right effects (evaluation scheme $E_{e}$) indicates that the ability to predict the right effects for an interacting drug–drug pair depends on which pair it is.

The values of the regularization parameters of the three-step model were tuned separately for optimal $E_{e}$ and $E_{dd}$ performance on training data, for both AUC-ROC and AUC-PR. Information on the tuned values, their link with the task, and corresponding optimal performance can be found in Appendix B.

Our model for prediction task $\hat{dde}$ and evaluation scheme $E_{dd}$ can be compared to the current state-of-the-art, as it is the only setting that has been tackled before. The best performing state-of-the-art model obtained AUC-ROC =0.965 and AUC-PR =0.938 [22,23,26]. We see that our AUC-ROC of 0.957 in that setting is quite competitive. The comparison of AUC-PR values is a bit more complicated. In our work, the full data set with intrinsic class imbalance of 0.02 was used for evaluation, resulting in a no-skill value of 0.02, whereas in previous work, the data was sampled in a balanced way such that the no-skill value was put to 0.5 (although it was suggested to use the intrinsic value of 0.02) [26].

In order to compare, we recalculated our result to that very setting, obtaining AUC-PR =0.957, which again is a comparable result. More details about the computation of AUC-PR can be found in Appendix D. We conclude that the three-step model is competitive with the current state-of-the-art for this specific setting, taking into account that we used full 10-fold cross-validation with stable results, whereas in published research only a single train-test split is used and performance may be more subject to statistical variations. Important to mention here is that this 10-fold cross-validation was only made feasible due to the availability of an algebraic solution and shortcuts for the three-step model [36].

### 2.4. Detecting New Adverse Drug-Drug Interaction Effects

**Figure 5:**
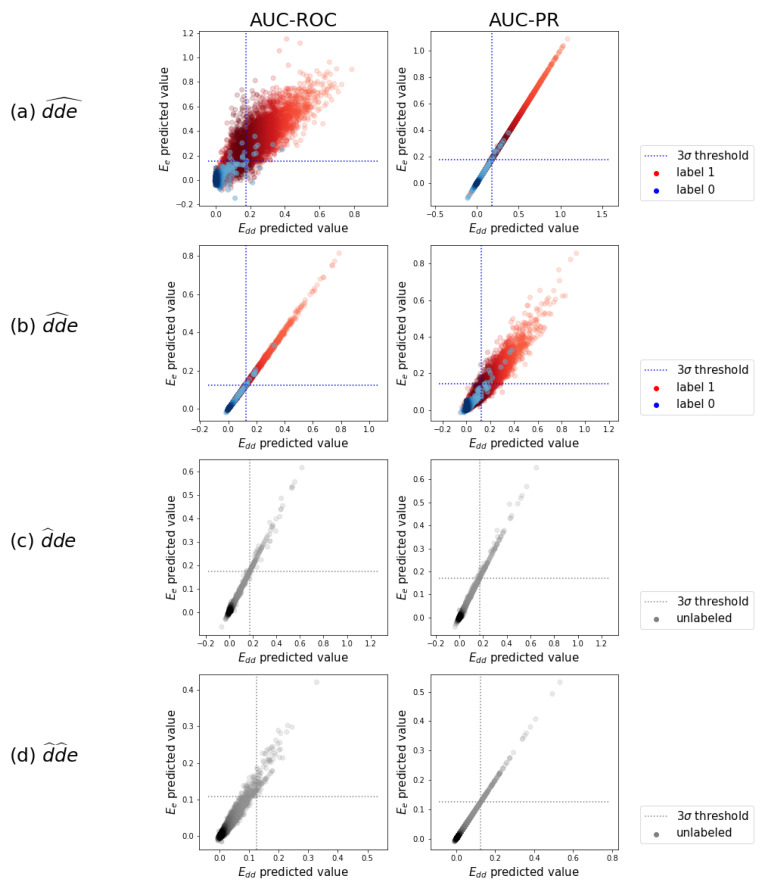
The test predictions. A random subsample of the test predictions of the $E_{e}$-optimized model versus the ones of the $E_{dd}$-optimized model are displayed. These are gathered from the ten test folds. (**a**) $\hat{dde}$ predictions in separate distributions for original zero-labels and one-labels, with a 3σ-threshold on the zero-label distribution. (**b**) Idem as in (**a**) for $\hat{dd}e$. (**c**) $\hat{d}de$ predictions as one distribution, since a label is never expected. (**d**) Idem as in (**c**) for $\hat{d}\hat{d}e$.

We illustrate how adverse drug–drug interaction effects can be detected using the test predictions of the above validated models. Figure 5a displays a random subsample of the task $\hat{dde}$ test predictions of the model optimized for $E_{dd}$ versus those of the one optimized for $E_{e}$. We observe that the predictions are strongly correlated. This correlation is not always obvious, as good predictions in one scheme may be useless in the other. Individual $E_{dd}$ and $E_{e}$ prediction histograms for each of the prediction tasks can be found in Appendix C.

For the triplets that were originally assigned a zero label, indeed, the highest density in predicted values is at zero in Figure 5a. A smaller number of predictions are pushed towards larger values. By setting a threshold, a subset of the larger predicted values can be selected as newly detected occurring triplets. We set this threshold at three times the standard deviation of the distribution centered near zero and do that for the four models that were tuned for both evaluation schemes and both AUC-ROC and AUC-PR. We obtain a set of newly detected triplets that contains 1.42% of all possible triplets. These are divided over 41,614 distinct drug–drug pairs, of which 99.9% of the pairs had already one or more other known effects in the data. This is what a task-one model is trained to do: predict additional effects when some others are already known. The other 0.1%, i.e., eleven pairs, are completely new interacting drug–drug combinations with adverse effects, which can be regarded as highly likely given that even a task-one model detected them, instead of a task-two model. More examples can be found in the Supplementary Material.

A similar analysis can be done for task $\hat{dd}e$, with predictions and thresholds shown in Figure 5b. We obtain a set of newly detected triplets that contains 1.48% of all possible triplets. These are divided over 74,693 distinct drug–drug pairs. Roughly 70% of these pairs cover 81% of drug–drug pairs that had at least one known other effect in the labeled data, without using it during training. The other 30% of these drug–drug pairs are newly predicted interacting combinations with adverse effects. More examples can be found in the Supplementary Material. One example of the new pairs that also could be confirmed afterwards by an external source (i.e., https://www.drugs.com/drug_interactions.html, accessed on 1 May 2021) is the interaction between compound CID000004999 (Quazepam) and compound CID000003559 (Haloperidol), leading to the effects “arterial pressure NOS decreased” and “hallucination” according to our model.

The analysis of the predictions in tasks $\hat{d}de$ and $\hat{d}\hat{d}e$ is somewhat different as it involves one or two drugs that are simulated as new drugs. We thus pretend that no interaction is known at all, and predictions are plotted as a single distribution in Figure 5c,d. We again set a threshold of three times the standard deviation. We obtain a set of triplets with predicted values above the threshold, which can be considered as most likely and could be investigated or tested in clinical trials. To evaluate the relevance of the set of most likely predicted triplets, we look back to the known interactions in the labeled data that we just neglected. For task $\hat{d}de$, 73% of the pairs in the predicted set effectively are interacting pairs with multiple adverse effects. This means that investigating the highest predictions by the model when a new drug is developed, would reveal an interacting combination in almost three out of four cases. For task $\hat{d}\hat{d}e$, 67% of the above-threshold predicted interacting pairs can be confirmed the data.

## 3. Discussion

Effect-specific prediction of adverse drug–drug interactions is highly relevant for pharmacovigilance. To this end, we fed a machine learning model with data mined from a post-market surveillance system. We distinguished between four prediction tasks of which two tasks aim to improve the surveillance system by detecting unknown additional adverse effects from drug–drug interactions, and two tasks that predict adverse interaction effects earlier on in the drug development process by considering one or two drugs that are new and thus not contained in the surveillance system.

We showed that each of the tasks has its own level of difficulty. The first one is a regular tensor completion problem, the other ones being cold-start tasks. We argued for fair model validation, each task requires its own cross-validation scheme, and we presented two different evaluation schemes, one focusing on predicting the right interacting drug–drug pairs, and another one focusing on predicting the right effects.

We introduced a model that can tackle all prediction tasks in a unified way. To solve these problems, we used side information on drugs in the form of single-drug effects as they indicate on which target a single drug is acting. However, more detailed side information could be included in the future to improve the prediction performance. Examples are explicit target proteins, the chemical structure of the drug, and so on.

Our models were trained and validated on the data published by Zitnik et al. [22], originating from adverse event reporting systems and containing 645 drugs and a variety of 963 effects. In the regular tensor completion task, performance is competitive with the state-of-the-art. More importantly, our approach can handle the new and more difficult cold-start tasks as well. As an illustration, we used the predictions of the validated models for our first two tasks and selected, by means of a 3σ-threshold, a list of additional adverse effects for known interacting drug–drug pairs and a list of new adverse drug–drug combinations. For the two latter tasks, we also made a selection based on predictions exceeding a 3σ-threshold, and observed that on average 73% of the selected adverse combinations for a new drug can indeed be confirmed to have at least one adverse effect, while in the case of two new drugs, this proportion was 67%.

The discussion on prediction tasks, model validation and the proposed model for predicting links between two drugs and an effect may be valid for a broader set of problems that can be considered as triplet link prediction. Obviously, it is also valid for modeling the positive effects and instead search for adequate treatments with drug combinations [21,27,28,29]. As drug effects depend on the patient, one could also predict patient-specific drug–drug interaction [37] with a drug–drug-patient triplet link, or predict patient-specific effects [38] with a patient-drug-effect triplet. More examples are tri-genic interactions [39] or context-dependent binary links such as tissue-dependent protein-protein interaction or tissue-dependent protein-function association [40].

## 4. Materials and Methods

### 4.1. Three-Step Kernel Ridge Regression

We propose the three-step kernel ridge regression model that can solve all of the identified prediction tasks. Recall that a kernel function takes as input two objects and returns a similarity score, taking higher values if the objects are more similar. Assume arbitrary kernel functions $k_{d}$ and $k_{e}$ that take as input two drugs or two effects, respectively. With $D=\{d_{i}|i=1,\ldots ,n_{d}\}$ the drugs and $E=\{e_{i}|i=1,\ldots ,n_{e}\}$ the effects that are present in the reporting system, the prediction for any new combination $\left(d,d^{\prime },e\right)$ is given by

(1) $$f\left(d,d^{\prime },e\right)=\sum_{a=1}^{n_{d}}\sum_{b=1}^{n_{d}}\sum_{c=1}^{n_{e}}A_{abc}k_{d}\left(d,d_{a}\right)k_{d}\left(d^{\prime },d_{b}\right)k_{e}\left(e,e_{c}\right)\;.$$

Here, $A_{abc}$ represents the tensor of model parameters that needs to be learned and must satisfy, for any i,j,k,

(2) $$Y_{ijk}=\sum_{a=1}^{n_{d}}\sum_{b=1}^{n_{d}}\sum_{c=1}^{n_{e}}A_{abc}\left(k_{d}\left(d_{i},d_{a}\right)+\lambda_{1}\delta_{ia}\right)\left(k_{d}\left(d_{j},d_{b}\right)+\lambda_{2}\delta_{jb}\right)\left(k_{e}\left(e_{k},e_{c}\right)+\lambda_{3}\delta_{kc}\right)\;.$$

This equation expresses that the label tensor must equal the predictions for a slightly adapted prediction function. The adaptation involves adding a small value $\lambda_{1}$, $\lambda_{2}$ or $\lambda_{3}$ to the similarity between two identical objects. These parameters are the regularization parameters of the model. The regularization prevents the model from overfitting, and instead allows it to improve predictions for triplets that are not present in the label tensor [30].

This model can solve the various cold-start problems in a unified way by assigning appropriate values to the regularization parameters. Each of them corresponds to the regularization strength for one of the three objects of the triplet. As an example, one could already foresee that in prediction task with a new drug in the first position, a higher $\lambda_{1}$ than $\lambda_{2}$ is to be used.

Another advantage of this model is efficiency. There exists an algebraic solution for the model parameters consisting of performing three tensor contractions such that finding the solution is guaranteed in a limited time. Besides, due to the linear structure, one can derive shortcuts for the cross-validation procedures [36]. Instead of training and evaluating a model for every fold explicitly, cross-validation can be done within the time complexity of a single training. The algebraic solution and shortcuts are discussed more in-depth in the Appendix A.

### 4.2. Data Set

The drug–drug interaction data set is downloaded from http://snap.stanford.edu/decagon/ [41] (accessed on 12 June 2020) and filtered as described in the work of Zitnik et al. [22]. The interaction data originates from the TWOSIDES project [11], where drug–drug interaction effect triplets were mined from adverse event reporting systems, while correcting for confounding factors. The TWOSIDES data set contained 1318 effect types across 63,473 drug combinations, where the effect is stronger than the expected effect of the drugs individually. This data was filtered by Zitnik [22] focusing on the most commonly occurring types of effects in at least 500 drug combinations. The resulting data set contains 645 drugs, which are molecules indexed by a PubChem CID number, and 963 adverse effects ranging from nausea, headache, or nightmare to acute kidney failure, lung fibrosis, heart attack, or still birth. 2% of the possible drug–drug-effect triplets represent an occurring effect, caused by 70% of all possible drug–drug pairs. Any other triplet is assumed to have a zero label.

Additionally, we also downloaded mono-drug effects in order to use them as side features for individual drugs (http://snap.stanford.edu/decagon/ [41], accessed on 12 June 2020). This data set was constructed from the SIDER database and OFFSIDES database [11]. By construction, the mono-drug effects in this set do not overlap with the drug–drug interaction effects, and thus can safely be used as side information without including label information.

### 4.3. Kernel Construction

We propose easy and straightforward strategies to compute the similarity kernel for the drugs, $k_{d}:D\times D\to R$, and the one for the interaction effects, $k_{e}:E\times E\to R$. For the similarity between two drugs, we use the mono-drug effects published by Zitnik et al. We define the kernel function as $k_{d}\left(d_{i},d_{j}\right)=\exp\left(-\left|\right|d_{i}-d_{j}{\left|\right|}^{2}/\gamma \right)$, with $d_{i}$ a vector of length γ that holds value 1 if a mono-drug effect occurs and 0 otherwise. For the similarity between effects, we do not use an external data source, but we use the training labels instead. The kernel function for the effects is given by the Tanimoto similarity $k_{e}\left(e_{i},e_{j}\right)=\frac{e_{i}\cdot e_{j}}{\left|\right|e_{i}\left|\right|\cdot \left|\right|e_{j}\left|\right|}$, with $e_{i}$ a vector that holds value 1 if it is caused by a certain train drug–drug pair and 0 otherwise. In all of the prediction tasks, there is a clear boundary between test and train drug–drug pairs, except for setting $\hat{dde}$, where every triplet is randomly assigned to a test fold. This little problem can be solved with a simple trick. Half of the $\hat{dde}$ folds use half of the drug–drug pairs, and the same for the other half, however, in theory this may worsen the performance a little bit. This strategy of using labels of the training data to construct kernels is not new and quite effective: the features are already engineered in such a way that they are likely to contain information on the (training) labels [42].

### 4.4. Experimental Setup and Tuning of Regularization Parameters

The 10-fold cross-validation is exactly performed as presented in Figure 2b, with a small modification for prediction task $\hat{dde}$ to be able to construct kernels for effects, as explained above. Within each training fold, the regularization parameters of the three-step model are tuned separately for each prediction task, for both evaluation schemes and for both performance metrics, by performing nested cross-validation for various values of the regularization parameters and selecting the best performing ones. This additional cross-validation is efficiently done using the leave-out cross-validation shortcuts of the model (see Appendix A). The training set is for every object very similar to the complete set since only one object is left out, making leave-one-out cross-validation very suitable for regularization parameter tuning. In tasks $\hat{dde}$, $\hat{dd}e$ and $\hat{d}\hat{d}e$, the role of both drugs is the same and we assumed $\lambda_{1}=\lambda_{2}$. We varied $\lambda_{1}$ from $10^{-6}$ to $10^{-1}$ and $\lambda_{3}$ from $10^{-4}$ to $10^{1}$ in $\hat{dde}$ and $\hat{d}de$; and $\lambda_{1}$ from $10^{-4}$ to $10^{1}$ and $\lambda_{3}$ from $10^{-4}$ to $10^{1}$ in $\hat{d}\hat{d}e$. In setting $\hat{d}de$, the role of both drugs is different and we varied $\lambda_{1}$ from $10^{-3}$ to $10^{-1}$, $\lambda_{2}$ from $10^{-6}$ to $10^{-1}$, and $\lambda_{3}$ from $10^{-1}$ to $10^{1}$. As expected, the optimal values depend on the setting, e.g., in setting $\hat{d}de$ a higher $\lambda_{1}=0.1$ and lower $\lambda_{2}=0.00001$ is found. Further, the evaluation scheme may have a slight influence on the optimal value, e.g., in $E_{e}$, where one wants to discriminate between effects, a smaller value of $\lambda_{3}$ for regularization for the effects is better. More detailed results and discussion on the optimal values for the regularization parameters can be found in Appendix B.

The predictions for the different test folds are pooled together for evaluation. This ensures that for each of the prediction tasks, only one $E_{dd}$ evaluation per effect and only one $E_{e}$ evaluation per drug–drug pair is computed leading to unambiguous distributions. If performance could be computed for each test fold separately, then we would end up with ten distributions for which aggregation for the various cross-validation schemes would complicate interpretation.

## 5. Conclusions

We formulated the problem of data-driven prediction of drug–drug interaction effects as triplet link prediction between two drugs and an effect caused by their interaction. Distinguishing between four different subtasks with a different level of “cold start”, depending on which other drug–drug interaction effects are known for the drugs is crucial. We introduced a model called the three-step kernel ridge regression which can efficiently solve the different tasks in a unified way. We also discussed validation procedures that are crucial to correctly assess the performance for various tasks.

Although the discussion is also valid for modeling positive drug–drug interaction effects, we focused on adverse interaction effects by using data extracted from adverse event reporting systems in the context of pharmacovigilance. Our model obtained AUC-ROC =0.957 for the easiest task, which is comparable to the state-of-the-art, but more importantly, could also solve the other cold-start tasks with AUC-ROC =0.843 for the hardest task that involves two newly developed drugs that are not yet present in pharmacovigilance systems.

We conclude that machine learning models, if combined with appropriate model validation for the desired task, could provide a highly relevant tool for predicting drug–drug interactions in pharmacovigilance as well as in the search for effective treatments.

## Figures and Tables

**Table 1:** Final test performances with pooling aggregation. These represent the averages of the distributions shown in Figure 4. They are obtained as illustrated in Figure 3 for both evaluation schemes. The test predictions of the ten folds were pooled together into one final evaluation such that each of the distributions is unambiguously determined. No-skill performance for AUC-ROC and AUC-PR is 0.5 and 0.02, respectively. Details on the computation and the no-skill value of AUC-PR can be found in Appendix D. Both metrics have perfect-skill performance at 1.

	AUC-ROC		AUC-PR	
	(No-Skill = 0.5)		(No-Skill = 0.02)	
	$E_{\mathrm{dd}}$	$E_{e}$	$E_{\mathrm{dd}}$	$E_{e}$
$\hat{dde}$	0.957	0.888	0.557	0.257
$\hat{dd}e$	0.919	0.865	0.286	0.179
$\hat{d}de$	0.910	0.859	0.221	0.176
$\hat{d}\hat{d}e$	0.843	0.834	0.112	0.144

**Figure A1:**
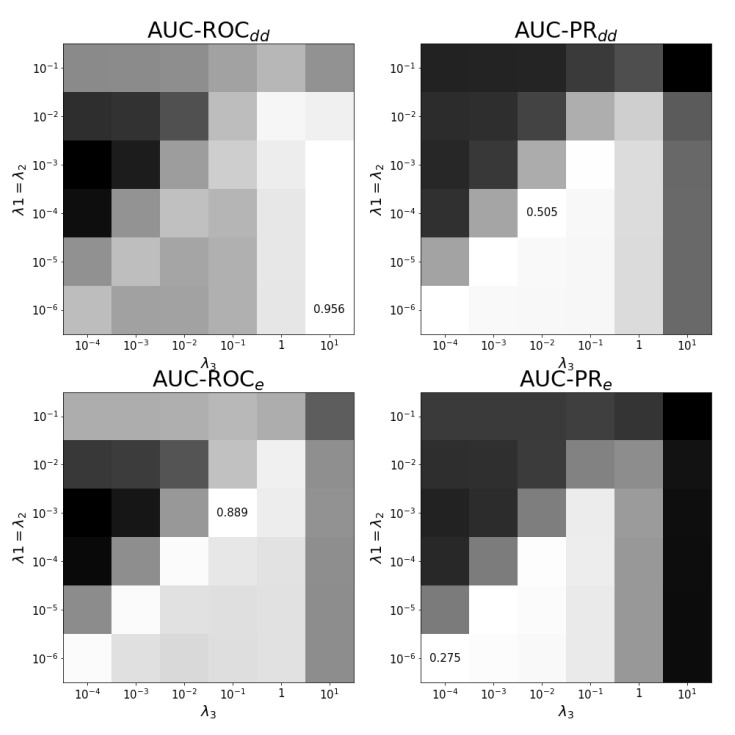
Tuning regularization parameters for task $\hat{dde}$. The average performance on ten training folds using shortcuts for leave-out validation is shown for different combinations of regularization parameters. The optimal combination and values are shown. White corresponds to the highest performance and black to the lowest performance. Each panel has a different color scale as performance varies over different scales.

**Figure A2:**
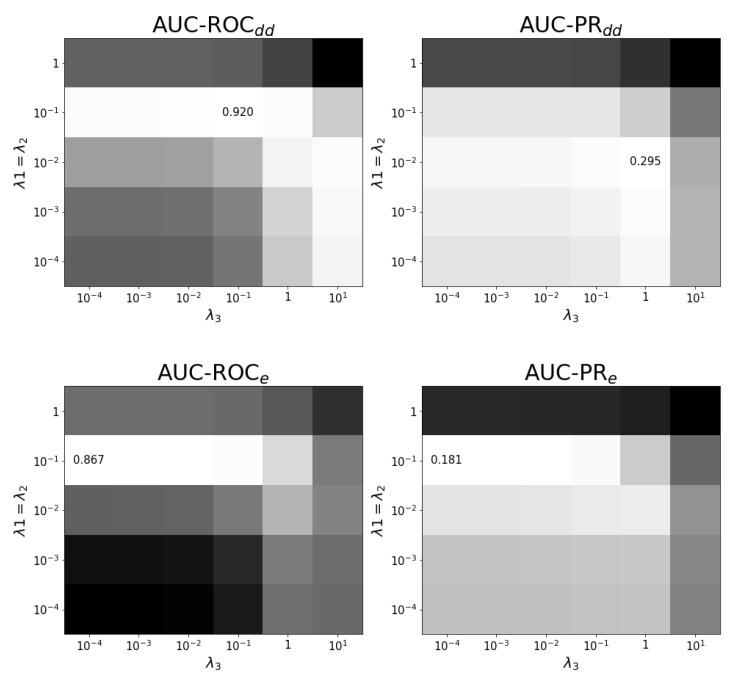
Tuning regularization parameters for task $\hat{dd}e$. The average performance on ten training folds using shortcuts for leave-out validation is shown for different combinations of regularization parameters. The optimal combination and values are shown. White corresponds to the highest performance and black to the lowest. Each panel has a different color scale as performance varies over different scales.

**Figure A3:**
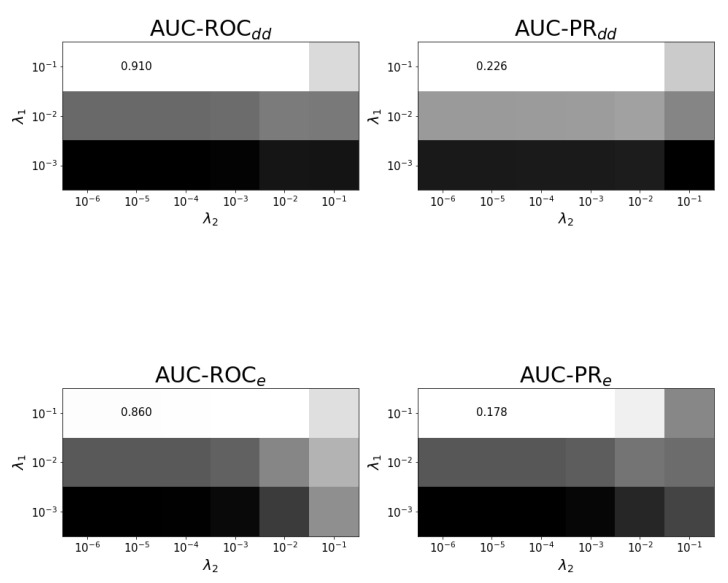
Tuning regularization parameters for task $\hat{d}de$ at optimal $\lambda_{3}$. The average performance on ten training folds using shortcuts for leave-out validation is shown for different combinations of regularization parameters. The optimal combination and values are shown. White corresponds to the highest performance and black to the lowest performance. Each panel has a different color scale as performance varies over different scales.

**Figure A4:**
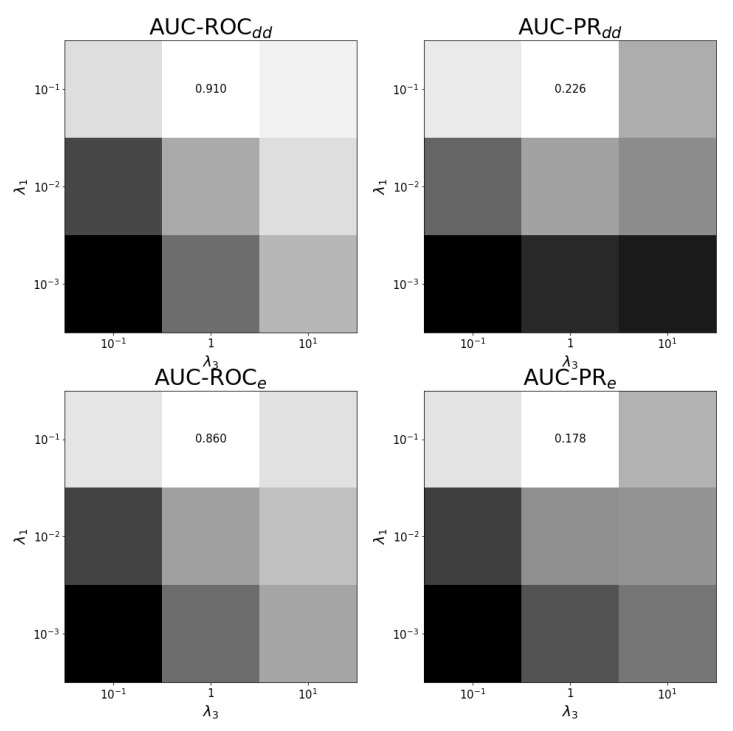
Tuning regularization parameters for task $\hat{d}de$ at optimal $\lambda_{2}$. The average performance on ten training folds using shortcuts for leave-out validation is shown for different combinations of regularization parameters. The optimal combination and values are shown. White corresponds to the highest performance and black to the lowest. Each panel has a different color scale as performance varies over different scales.

**Figure A5:**
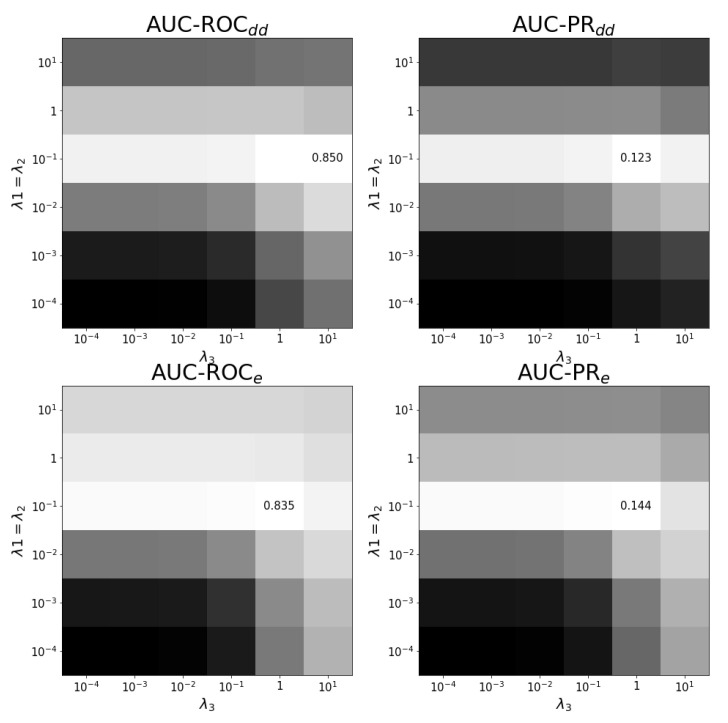
Tuning regularization parameters for task $\hat{d}\hat{d}e$. The average of performance on ten training folds using shortcuts for leave-out validation is shown for different combinations of regularization parameters. The optimal combination and values are shown. White corresponds to the highest performance and black to the lowest performance. Each panel has a different color scale as performance varies over different scales.

**Figure A6:**
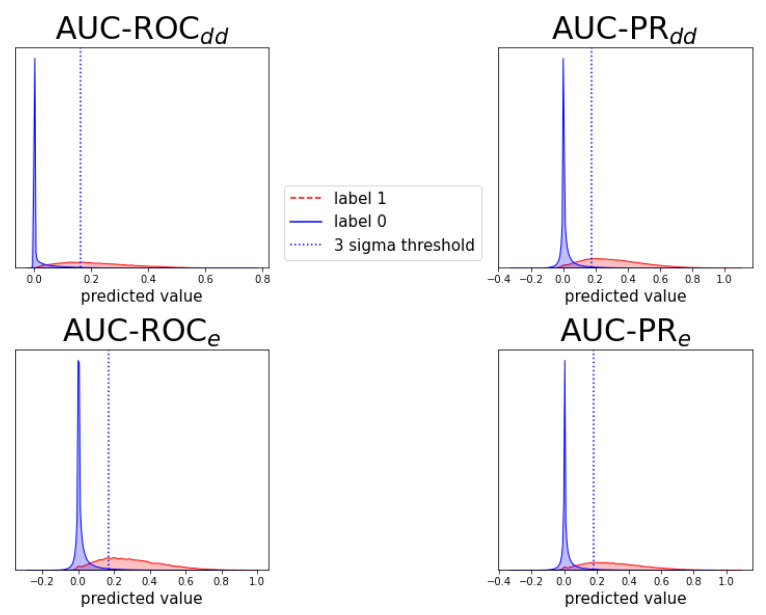
Individual prediction histograms of the model for task $\hat{dde}$. The predictions for the ten test folds are pooled and plotted separately for the originally zero-labeled and one-labeled triplets. For zero-labeled triplets, a prediction peak near zero is found and a 3σ-threshold is shown. Each of the panels shows the results of a model optimized for AUC-ROC or AUC-PR and evaluation scheme $E_{dd}$ or $E_{e}$.

**Figure A7:**
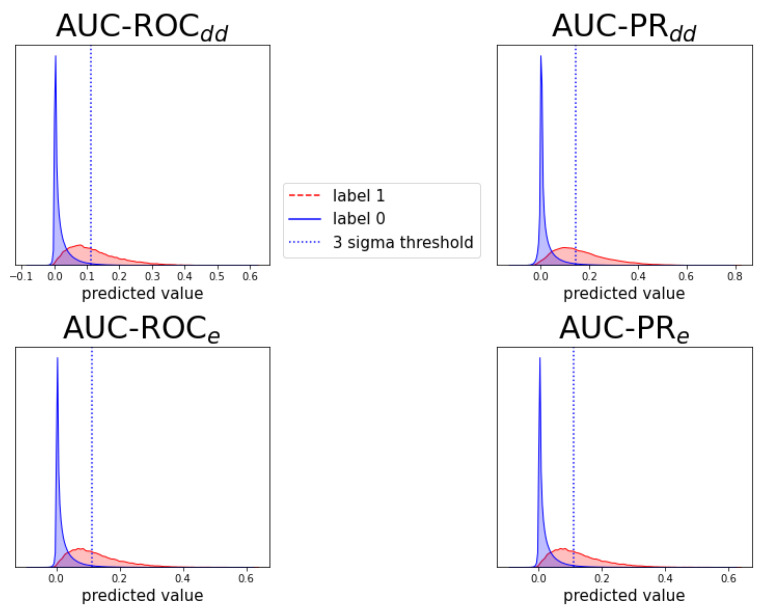
Analogous to Figure A6, for prediction task $\hat{dd}e$.

**Figure A8:**
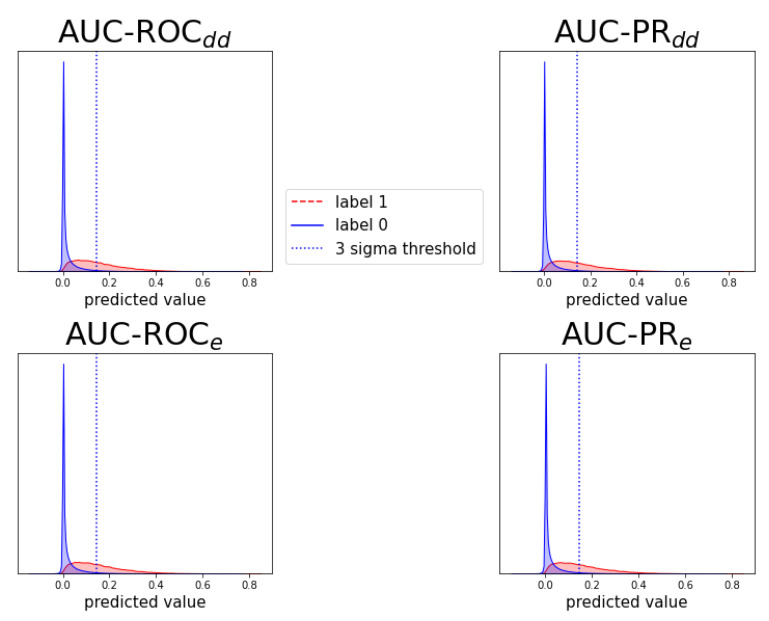
Analogous to Figure A6, for prediction task $\hat{d}de$.

**Figure A9:**
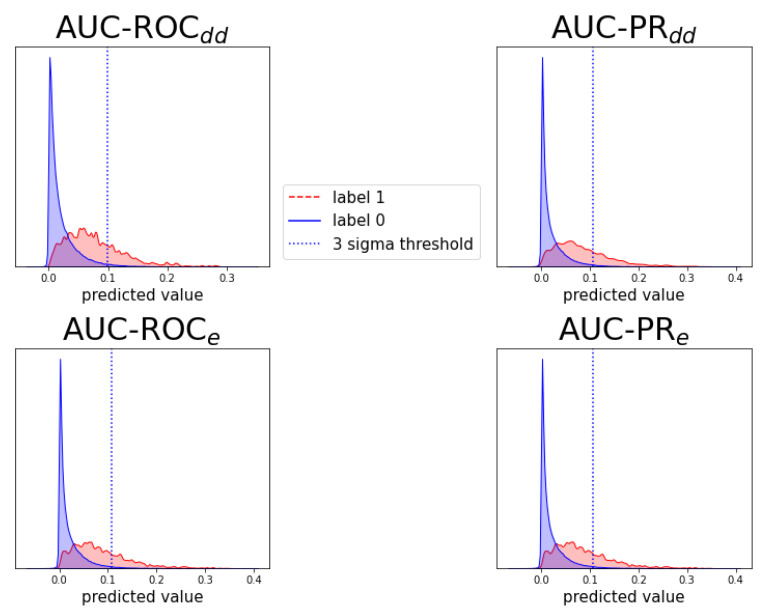
Analogous to Figure A6, for prediction task $\hat{d}\hat{d}e$.

## Data Availability

The relevant data is available via references in the text. Research code can be made available for a publication in a publicly accessible repository.

## Supplementary Materials

The following are available online at https://www.mdpi.com/article/10.3390/ph14050429/s1.

## Appendix A. Three-Step Kernel Ridge Regression: Algebraic Expressions for Model Parameters and Cross-Validation Shortcuts

We present three-step kernel ridge regression as a generalization of common kernel ridge regression. We first summarize the algebraic expressions for the common one and show how cross-validation shortcuts can be computed.

### Appendix A.1. Kernel Ridge Regression and Shortcuts

For a common one-dimensional regression training data set $\left\{\left(x_{i},y_{i}\right)|i=1,2,\ldots ,n\right\}$ with objects $x_{i}\in X$ and corresponding labels $y_{i}\in R$, and a kernel function k(·,·) that expresses similarity between objects *x* and $x^{\prime }$, the functional form for kernel ridge regression is written as

(A1) $$f\left(x\right)=\sum_{j=1}^{n}\alpha_{j}k\left(x,x_{j}\right)\;.$$

Here, $\alpha_{j}$ are the model parameters that need to be learned. By restricting predictions to the training set, using $f_{i}$ as a shorthand notation for $f(x_{i})$, and defining the kernel matrix *K* by function evaluations, i.e., $K_{ij}=k\left(x_{i},x_{j}\right)$, we can write

(A2) $$f_{i}=\sum_{j=1}^{n}\alpha_{j}K_{ij}\;.$$

Inserting this formula in the kernel ridge regression loss function with introduction of a ridge hyper-parameter λ yields the algebraic solution

(A3) $$\alpha_{i}=\sum_{j=1}^{n}B_{ij}y_{j}$$

with

$$B={\left(K+\lambda I\right)}^{-1}\;,$$

such that the predicted values for the training labels can be computed as

(A4) $$f_{i}=\sum_{j=1}^{n}H_{ij}y_{j}$$

with the hat matrix *H* defined as

$$H=K{\left(K+\lambda I\right)}^{-1}\;.$$

The hat matrix represents the linear mapping from the labels $y_{i}$ to the predictions $f_{i}$. This matrix enables us to efficiently calculate the leave-out value $f_{i}^{out}$ that does not use label $y_{i}$ [43]. The idea is to create a new set of labels where $y_{i}$ is replaced by $f_{i}^{out}$. Training on this new set of labels makes sure that $y_{i}$ is not included and this circular reasoning makes sure that $f_{i}^{out}$ is in equilibrium with the model: it is both the observed and the predicted value:

$$f_{i}^{out}=\left(\sum_{j=1}^{n}H_{ij}y_{j}\right)-H_{ii}y_{i}+H_{ii}f_{i}^{out}\;.$$

Here, the first term is the expression as before, whereas the second and third terms cancel the contribution of $y_{i}$ and replace it by a contribution of $f_{i}^{out}$, respectively. This equation can be rewritten as

(A5) $$f_{i}^{out}=\sum_{j=1}^{n}O_{ij}y_{j}$$

where we see that to leave out element $y_{j}$, all we need to do is replacing *H* by *O*:

$$O_{ij}=\frac{H_{ij}-H_{ii}}{1-H_{ii}}\;,$$

i.e., subtract the diagonal elements and rescale. This will make sure that the prediction of $f_{i}^{out}$ is not influenced by the observation of $y_{i}$. We can thus compute the leave-out estimations for all the training data, and thus do a complete leave-out cross-validation with the same time complexity as computing the model parameters once.

### Appendix A.2. Three-Step Kernel Ridge Regression and Shortcuts

For the three-step version of kernel ridge regression, one needs a parameter tensor with three indices. It allows to do an independent ridge regression for each of the objects of the triplet, conditioned on the combination of the other two. Restricting the predictions to drugs and interaction effects from the training data, we define the tensor $F_{ijk}=f\left(d_{i},d_{j},s_{k}\right)$ that predicts the values of the label tensor $Y_{ijk}$. By defining the kernel matrices $K^{d}$ as ${K^{d}}_{ia}=k_{d}\left(u_{i},u_{a}\right)$ and $K^{e}$ as ${K^{e}}_{kc}=k_{s}\left(w_{k},w_{c}\right)$ as function evaluations for the similarity between the training drugs and the interaction effects, we can write the prediction function as

(A6) $$F_{ijk}=\sum_{a=1}^{n_{d}}\sum_{b=1}^{n_{d}}\sum_{c=1}^{n_{e}}A_{abc}{K^{d}}_{ia}{K^{d}}_{jb}{K^{e}}_{kc}\;.$$

This is the triple version of (A2), for which we compute the solution

(A7) $$A_{ijk}=\sum_{a=1}^{n_{d}}\sum_{b=1}^{n_{d}}\sum_{c=1}^{n_{e}}Y_{abc}G_{ia}^{\left(1\right)}G_{jb}^{\left(2\right)}G_{kc}^{\left(3\right)}$$

with

$$G^{\left(1\right)}={\left(K^{d}+\lambda_{1}I\right)}^{-1}$$

$$G^{\left(2\right)}={\left(K^{d}+\lambda_{2}I\right)}^{-1}$$

$$G^{\left(3\right)}={\left(K^{e}+\lambda_{3}I\right)}^{-1}$$

as generalization of Equation (A3), giving rise to following expression for the predicted values

(A8) $$F_{ijk}=\sum_{a=1}^{n_{d}}\sum_{b=1}^{n_{d}}\sum_{c=1}^{n_{e}}H_{ia}^{\left(1\right)}H_{jb}^{\left(2\right)}H_{kc}^{\left(3\right)}Y_{abc}$$

with

$$H^{\left(1\right)}=K^{d}{\left(K^{d}+\lambda_{1}I\right)}^{-1}$$

$$H^{\left(2\right)}=K^{d}{\left(K^{d}+\lambda_{2}I\right)}^{-1}$$

$$H^{\left(3\right)}=K^{e}{\left(K^{e}+\lambda_{3}I\right)}^{-1}$$

as generalization of Equation (A4). Again, this represents a linear mapping form the observed labels to the predicted ones, allowing us to transform the mapping to leave certain observed labels out, according to the cross-validation setting.

The cross-validation shortcuts for the different settings are given by

(A9) $$F_{ijk}^{\hat{d}de}=\sum_{a=1}^{n_{d}}\sum_{b=1}^{n_{d}}\sum_{c=1}^{n_{e}}O_{ia}^{\left(1\right)}H_{jb}^{\left(2\right)}H_{kc}^{\left(3\right)}Y_{abc}$$

(A10) $$F_{ijk}^{\hat{d}\hat{d}e}=\sum_{a=1}^{n_{d}}\sum_{b=1}^{n_{d}}\sum_{c=1}^{n_{e}}O_{ia}^{\left(1\right)}O_{jb}^{\left(2\right)}H_{kc}^{\left(3\right)}Y_{abc}$$

(A11) $$F_{ijk}^{\hat{dd}e}=\sum_{a=1}^{n_{d}}\sum_{b=1}^{n_{d}}\sum_{c=1}^{n_{e}}O_{ia;jb}^{\left(12\right)}H_{kc}Y_{abc}$$

(A12) $$F_{ijk}^{\hat{dde}}=\sum_{a=1}^{n_{d}}\sum_{b=1}^{n_{d}}\sum_{c=1}^{n_{e}}O_{ia;jb;kc}^{\left(123\right)}Y_{abc}\;,$$

with

$$O_{ij}^{\left(1\right)}=\frac{H_{ij}^{\left(1\right)}-H_{ii}^{\left(1\right)}}{1-H_{ii}^{\left(1\right)}}$$

$$O_{ij}^{\left(2\right)}=\frac{H_{ij}^{\left(2\right)}-H_{ii}^{\left(2\right)}}{1-H_{ii}^{\left(2\right)}}$$

$$O_{ia;jb}^{\left(12\right)}=\frac{H_{ia}^{\left(1\right)}H_{jb}^{\left(2\right)}-H_{ii}^{\left(1\right)}H_{jj}^{\left(2\right)}}{1-H_{ii}^{\left(1\right)}H_{jj}^{\left(2\right)}}$$

$$O_{ia;jb;kc}^{\left(123\right)}=\frac{H_{ia}^{\left(1\right)}H_{jb}^{\left(2\right)}H_{kc}^{\left(3\right)}-H_{ii}^{\left(1\right)}H_{jj}^{\left(2\right)}H_{kk}^{\left(3\right)}}{1-H_{ii}^{\left(1\right)}H_{jj}^{\left(2\right)}H_{kk}^{\left(3\right)}}\;.$$

These shortcuts can be explained as follows:

- $\hat{d}de$. As $H^{1}$ performs the regression for the first drug, we can simply replace $H^{1}$ by its leave-out variant $O^{1}$. This makes sure that predictions for a certain first drug do not use the labels for that drug.
- $\hat{d}\hat{d}e$. A similar reasoning applies, but now $H^{1}$ and $H^{2}$ are replaced by $O^{1}$ and $O^{2}$, respectively.
- $\hat{dde}$. The same strategy is followed, however, the situation is somewhat more difficult: the operation of subtracting the diagonal elements of the hat matrix and rescaling must now be applied on the combined tensor product of the matrices, instead of aplying it separately. Therefore, $H_{ia}H_{jb}H_{kc}$ is replaced by a combined $O_{ia;jb;kc}$.
- $\hat{dd}e$. The same strategy is followed, however, the situation is somewhat more difficult: the operation of subtracting the diagonal elements of the hat matrix and rescaling must now be applied on the combined tensor product of the two drug matrices, instead of applying it separately. Therefore, $H_{ia}H_{jb}$ is replaced by a combined $O_{ia;jb}$.

## Appendix B. Results on Hyper-Parametertuning

The performance results on the training data using the shortcuts for hyper-parameter tuning are averaged across ten folds and shown in Figure A1, Figure A2, Figure A3, Figure A4 and Figure A5.

In general, the optimal regularization parameters have the smallest values in $\hat{dde}$, see Figure A1. Note that, for AUC-ROC${}_{dd}$, very small regularization values are selected for the drugs, while very large ones are selected for the effects, corresponding to a model that is very specific for the drugs, but predicts nearly the same values for the different effects. This is not the case in the AUC-ROC${}_{e}$ scheme, where there is an optimal ratio between both hyper-parameters visible in the optimal diagonal. Note that for this task, it is rather the ratio between the hyper-parameters that is important.

In prediction task $\hat{dd}e$, see Figure A2, the regularization parameter for the drugs is rather fixed, as the model needs to correctly generalize to unseen drug–drug pairs. Further, there is an optimal value for regularization parameter $\lambda_{3}$ for the side effects, which is smaller in the $E_{e}$ evaluation scheme than in the $E_{dd}$ evaluation scheme: in the $E_{e}$ scheme, the model needs to be more specific for the effects.

In prediction task $\hat{d}de$, see Figure A3, the role of both drugs is different because the first drug is seen as a new drug and $\lambda_{1}$ and $\lambda_{2}$ are varied separately. Figure A3 shows that indeed $\lambda_{1}$ needs to have a larger value than $\lambda_{2}$ to generalize to new drugs. Figure A4 shows the dependence of performance on $\lambda_{1}$ and $\lambda_{3}$ for the effects.

Finally, in prediction task $\hat{d}\hat{d}e$, see Figure A5, bot the optimal values for the drug and effect regularization parameters have again a relatively high value. This model needs the most regularization of all models.

## Appendix C. Individual Prediction Histograms

See Figure A6, Figure A7, Figure A8 and Figure A9.

## Appendix D. Notes on Computation of Auc-Pr in Evaluation Schemes $E_{e}$ and $E_{\mathrm{Dd}}$

The AUC-PR score is a metric often used in case of class imbalance and focuses more on correctly predicting the minority class. Further, the value of this metric is sensitive to the ratio between the minority and majority class: while the perfect-skill score remains always at one, the no-skill score equals that ratio [44]. This complicates comparing different AUC-PR scores if the ratio is different. Two ways exist to account for this effect. One is sampling the label data according to the desired ratio and then evaluating the metric. Another one is by computing precision and recall on the full set of labels and performing an algebraic transformation that allows to compute the AUC-PR score with the desired virtual ratio. When calculating AUC-PR in $E_{dd}$ or $E_{e}$, the ratio of positive and negative labels may fluctuate depending on the effect or the drug–drug pair, see Figure 3. To obtain a consistent no-skill, we rescale every AUC-PR score to a virtual ratio that is the average ratio of the entire data set, i.e., 0.02. Previous research in this area put this no-skill virtually at 0.5 by sampling data in a balanced way, however possibly indicating optimistic results [26]. To compare with such results, we use the algebraic transformation [44] to rescale our results to the no-skill value of 0.5.
