# Supplementary material for: Cold-Start Problems in Data-Driven Prediction of Drug–Drug Interaction Effects
Source: Pharmaceuticals (Basel). 2021 May 2;14(5):429. doi: 10.3390/ph14050429 (PMC8147651; doi:10.3390/ph14050429)
Supplement: Supplementary file 1 [file pharmaceuticals-14-00429-s001.zip › pharmaceuticals-1173669-suppl.pdf]

This supplementary material provides some examples of predictions of the model that are discussed in Section 2.4. This includes predictions for Task1 and Task2 for (I) interactions that could be confirmed by the test data and (II) interactions that could not be verified and that can be interpreted as newly detected combinations with adverse effects. Drugs are indexed by the CID PubChem compound index (https://pubchem.ncbi.nlm.nih.gov/).

# (I) Interaction check of predictions

## Task 1

The model is trained to predict additional interaction effects for drug-drug pairs if it already knows other interaction effects. Above-3σ predictions for test labels are selected as detected adverse effects. These are compared with the adverse effects in the true test labels. Effects were detected (3σ) in 42587 drug pairs. Matching effects are indicated in bold.

CID000004771 (Phentermine),CID000003339 (Fenofibrate)
Detected (3σ):

"['**tendon injury'**, **'abuse**', '**bursitis**', '**Tendinitis'**, **'sleep walking**']

True test labels:

"['drowsiness', 'Abnormal Gait', 'weight gain', 'arterial pressure NOS decreased', 'Amnesia', 'Hallucination', 'Fatigue', 'edema extremities', **'tendon injury'**, **'abuse**', 'eating disorder', '**Tendinitis**', 'drug withdrawal', '**sleep walking**', 'Anorexia', 'Aching joints', '**bursitis'**, 'Back Ache', 'Pain', 'muscle spasm']",

CID000005515,CID000000853 (DL-Thyroxine)
Detected (3σ):

"['**anaemia'**, 'diarrhea']"

True test labels:

"['Fatigue', 'Dyspnea exertional', 'constipated', 'asthenia', 'bone marrow failure', 'Blood calcium decreased', 'emesis', 'Head ache', 'thrombocytopenia', 'nausea', 'Pain', 'blood sodium decreased', 'Electrolyte disorder', 'Neutropenia', **'anaemia**', 'arterial pressure NOS decreased', 'Feeling unwell', 'heart rate increased', 'abdominal pain', 'Excess potassium', 'eruption', 'muscle weakness']"

CID000060843,CID000003749 (Irbesartan)
Detected (3σ):

"['**diarrhea**', '**Neutropenia**', **'body temperature increased'**, '**dehydration'**, '**neumonia**', '**bone marrow failure**', '**thrombocytopenia**', 'nausea', 'arterial pressure NOS decreased', 'AFIB', 'emesis', '**anaemia**']"

True test labels:

"['Bilirubinaemia', 'hypoxia', 'asthenia', 'Aspartate Aminotransferase Increase', 'Blood calcium decreased', **'thrombocytopenia'**, 'angiitis', 'arthropathy', 'Difficulty breathing', 'atelectasis', 'Near Syncope', 'Pain', '**diarrhea**', 'coughing blood', 'Amnesia', 'Anorexia', 'sepsis', 'arterial pressure NOS decreased', '**Neutropenia**', 'heart rate increased', 'hyperglycaemia', '**body temperature increased**', 'blood sodium

decreased', 'Hypotension Orthostatic', '**dehydration**', 'aspiration pneumonia', 'eruption', 'drowsiness', 'increased white blood cell count', 'respiratory failure', **'anaemia**', '**neumonia**', **'bone marrow failure'**]"

## Task 2

The model is trained to predict interaction effects for drug-drug pairs if it knows no other interaction effects. Above-3σ predictions for test labels are selected as detected adverse effects. These are compared with the adverse effects in the true test labels. Effects were detected (3σ) in 53232 drug pairs. Matching effects are indicated in bold. Drugs are indexed by the CID PubChem compound index.

CID000003062 (Lanicor),CID000003724 (Iodixanol)
Detected (3σ):
"['**kidney failure**', 'Extremity pain', 'nausea', 'emesis', 'asystole', **'acute kidney failure**']"
True test labels:
"[**'kidney failure**', 'Bleeding', **'acute kidney failure**', 'arterial pressure NOS decreased', 'dysuria']"

CID000000206 (Hexose),CID000004513 (Axid Ar)
Detected (3σ):
"['abdominal pain', 'arterial pressure NOS decreased', 'heart rate increased', 'nausea', 'Pleural Effusion', 'neumonia', 'thrombocytopenia', 'anaemia', 'respiratory failure', '**still birth**']"
True test labels:
['**still birth**']

CID000060754 (Gadodiamide),CID000000598 (2-Mercaptoethanesulfonic acid)
Predicted:
"['**pleural pain**', 'asystole', 'Extremity pain', '**xerosis**', 'cardiac valvulopathy', **'lung fibrosis'**, '**contracture**']"
True test labels:
"['**xerosis**', 'cardiac valvulopathy', '**pleural pain**', **'lung fibrosis'**, **'contracture'**, 'asystole', 'Extremity pain']",

CID000003062 (Lanicor),CID000060696
Detected (3σ):
['**cardiovascular collapse**']
True test labels:
"['Hepatic failure', '**cardiovascular collapse**', 'Interstitial nephritis', 'thrombocytopenia', 'asystole', 'tachycardia ventricular']"

CID000170361 (Varenicline),CID000000861 (Rathyronine)

Detected (3σ):

"['Hallucination', 'dizziness', 'hypoglycaemia', '**insomnia**', 'arterial pressure NOS decreased', 'Aching joints', 'Amnesia', 'confusion', 'tremor', 'constipated', '**nightmare**', 'edema extremities', 'abdominal pain upper', 'Abnormal Gait', 'nervous tension', **'pleural pain'**, 'hyperglycaemia', 'aching muscles', 'bulging', 'Anorexia', **'chest pain'**, 'Difficulty breathing', 'Fatigue', 'asthenia', 'loss of weight', **'Pain**', 'Head ache', 'blurred vision', 'agitated', '**Hypoventilation'**, 'dyspepsia', 'aptyalism', 'allergies', 'Cough', 'drowsiness', 'bronchitis', 'nausea', 'Anxiety', 'emesis', 'panic attack', 'Feeling unwell', 'sleep disorder', '**weight gain**', 'Back Ache']"

True test labels:

"[**'insomnia**', 'lung neoplasms', '**Hypoventilation**', **'nightmare', 'pleural pain'**, **'chest pain'**, 'attempted suicide', **'Pain'**, '**weight gain**']"

## (II) Interactions for drug-drug pairs that could not be verified: new adverse combinations

### Task 1

The Task 1 model was mainly trained to detect additional interaction effects based on other interaction effects. Exceptionally, it detected for 11 drug-drug pairs also new interaction effects, without any other interaction effect known. Examples are given below.

CID000001302,CID000002673
Detected (3σ):

"['Difficulty breathing', 'Anxiety', 'pleural pain', 'chest pain']"

CID000001302,CID000003562 (Halothane)
Detected (3σ):

"['chest pain', 'pleural pain', 'dizziness', 'Anxiety']"

CID000005052 (Isoreserpin),CID000001302
Detected (3σ):
"['Anxiety', 'insomnia', 'pleural pain', 'Difficulty breathing', 'chest pain']"

CID000005039 (Ranisen),CID000003562 (Halothane)
Detected (3σ)

"['Extremity pain', 'muscle spasm']"

### Task 2

The model is trained to predict interaction effects for drug-drug pairs if it knows no other interaction effects. Above-3σ predictions for test labels are selected as detected adverse effects. Effects were detected (3σ) in 23026 drug-drug pairs. These could not be confirmed by the data and are newly detected drug interactions.

CID000060843,CID000005379 (Gatifloxacin)
Detected (3σ):

"['Pleural Effusion', 'dehydration', 'thrombocytopenia', 'anaemia']"

CID000003261 (Estazolam),CID000002909 (Cyclosporine A)
Detected (3σ):

"['emesis', 'sepsis', 'body temperature increased', 'anaemia', 'acute kidney failure']"

CID000170361 (Varenicline),CID000003393 (Flurazepam)
Detected (3σ):

"['arterial pressure NOS decreased', 'nightmare', 'abdominal pain upper', 'muscle spasm', 'emesis', 'Difficulty breathing', 'nervous tension', 'asthenia', 'weight gain', 'Back Ache', 'sleep disorder', 'balance disorder', 'pleural pain', 'loss of consciousness', 'Amnesia', 'nausea', 'Hallucination', 'edema', 'convulsion', 'Abnormal Gait', 'abdominal pain', 'dizziness', 'edema extremities', 'Incontinence', 'confusion', 'drowsiness', 'Fatigue', 'bruxism', 'narcolepsy', 'insomnia', 'Anxiety', 'constipated', 'musculoskeletal pain', 'bipolar disorder', 'Head ache', 'Cough', 'Hypoventilation', 'allergies', 'Pain', 'hyperhidrosis', 'Anorexia', 'loss of weight', 'cognitive disorder', 'agitated', 'chest pain', 'Feeling unwell', 'hyperglycaemia', 'joint swelling', 'tremor', 'drug withdrawal', 'chronic obstructive airway disease']"

CID000003062 (Lanicor),CID000003168 (Droperidol)
Detected (3σ):

"['Difficulty breathing', 'Pain', 'body temperature increased', 'neumonia']"

CID005493444 (Aliskiren),CID000003016 (Diazepam)
Detected (3σ):

"['dizziness', 'asthenia', 'Difficulty breathing', 'arterial pressure NOS decreased']"
